# The Ear

**Published:** 1890-03

**Authors:** 


### THE EAR.

But few people thoroughly realize what a delicate structure the human ear really is.

That which we ordinarily designate so is, after all, only the mere outer porch of a series of winding passages which, like the lobbies of a great building, lead from the world without to the world within.

Certain of these passages are full of liquid and their membranes are stretched like parchment curtains across the corridor at different places, and can be thrown into vibrations or made to tremble like the head of a drum or as the surface of a tambourine does when struck with a stick or with the fingers.

Between two of these parchment-like curtains a chain of very small bones extends, which serves to tighten or relax these membranes and to communicate vibrations to them. In the innermost place of all a row of white threads, called nerves, stretchs like the strings of a piano from the last point to which the tremblings or thrillings reach and pass inward to the brain.

